# Volcanic Ash and Daily Mortality in Sweden after the Icelandic Volcano Eruption of May 2011

**DOI:** 10.3390/ijerph10126909

**Published:** 2013-12-10

**Authors:** Anna Oudin, Hanne K. Carlsen, Bertil Forsberg, Christer Johansson

**Affiliations:** 1 Division of Occupational and Environmental Medicine, Umeå University, Umeå 90187, Sweden; E-Mails: hannekcarlsen@envmed.umu.se (H.K.C.); bertil.forsberg@envmed.umu.se (B.F.); 2 Centre of Public Health Sciences, University of Iceland, Stapi v/Hringbraut, Reykjavík 101, Iceland; 3 Department of Applied Environmental Science, Stockholm University, Stockholm 10691, Sweden; E-Mail: christer.johansson@itm.su.se; 4 Environment and Health Administration, P.O. Box 38145, Stockholm 100 64, Sweden

**Keywords:** air pollution, particulate matter, volcano, mortality, ash, Iceland, Grimsvötn

## Abstract

In the aftermath of the Icelandic volcano Grimsvötn’s eruption on 21 May 2011, volcanic ash reached Northern Europe. Elevated levels of ambient particles (PM) were registered in mid Sweden. The aim of the present study was to investigate if the Grimsvötn eruption had an effect on mortality in Sweden. Based on PM measurements at 16 sites across Sweden, data were classified into an ash exposed data set (Ash area) and an unexposed data set (No ash area). Data on daily all-cause mortality were obtained from Statistics Sweden for the time period 1 April through 31 July 2011. Mortality ratios were calculated as the ratio between the daily number of deaths in the Ash area and the No ash area. The exposure period was defined as the week following the days with elevated particle concentrations, namely 24 May through 31 May. The control period was defined as 1 April through 23 May and 1 June through 31 July. There was no absolute increase in mortality during the exposure period. However, during the exposure period the mean mortality ratio was 2.42 compared with 2.17 during the control period, implying a relatively higher number of deaths in the Ash area than in the No ash area. The differences in ratios were mostly due to a single day, 31 May, and were not statistically significant when tested with a Mann-Whitney non-parametric test (*p* > 0.3). The statistical power was low with only 8 days in the exposure period (24 May through 31 May). Assuming that the observed relative differences were not due to chance, the results would imply an increase of 128 deaths during the exposure period 24–31 May. If 31 May was excluded, the number of extra deaths was reduced to 20. The results of the present study are contradicting and inconclusive, but may indicate that all-cause mortality was increased by the ash-fall from the Grimsvötn eruption. Meta-analysis or pooled analysis of data from neighboring countries might make it possible to reach sufficient statistical power to study effects of the Grimsvötn ash on morbidity and mortality. Such studies would be of particular importance for European societies preparing for future large scale volcanic eruptions in Iceland.

## 1. Introduction

In the aftermath of the Icelandic volcano Grimsvötn’s eruption on 21 May 2011, volcanic ash reached Northern Europe. Elevated levels of ambient particles (particulate matter, PM) were registered by ground level monitors in Sweden, Norway and Finland [1,2]. It is well known that PM can have acute effects on mortality and cardiorespiratory events, [3,4,5,6,7] but little is known about health effects of volcanic ash at similar doses.

Historically, Icelandic volcanos have affected Europe’s population severely; the most infamous example is the Laki volcano which erupted in 1783, causing thousands of deaths all over Europe [8]. More recently, ash from the Eyjafjallajökull eruption of 2010 caused massive delays in air-line traffic in large parts of Europe. The Grimsvötn volcanic eruption of May 2011 produced a large amount of ash in a short time compared to the 2010 Eyjafjallajökull eruption. Both the Eyjafjalajökull eruption of 2010 and the Grimsvötn eruption of 2011 are, however, small eruptions compared to the Laki eruption of 1783 and the historic eruptions of Katla, the perhaps most infamous of the Icelandic volcanoes. Katla is overdue for an eruption according to most estimates [9,10]. A Laki-style eruption has been estimated to cause up to 140,000 European deaths if it were to happen today [11], but that estimate was based on dose-response curves for ambient particles stemming from non-volcanic sources, such as traffic. It is highly uncertain if the use of dose-response curves based on traffic pollution would be valid to assess the potential health effects of volcanic ash exposures.

Although 9% of the world’s population reside within 100 km of an active volcano, [12] there are few epidemiological studies on health effects from particulate air pollution from volcanic eruptions [13,14]. It is thus urgent for health authorities to assess health effects of particles stemming from volcanic eruptions in order to rely on scientific knowledge when directing preventive measures in a scenario where high quantities of volcanic ash reach populated areas. The aim of the present study was to investigate if the ash from the Grimsvötn eruption had an effect on mortality in Sweden.

## 2. Material and Methods

### 2.1. Particle Concentration Measurements

**Figure 1:**
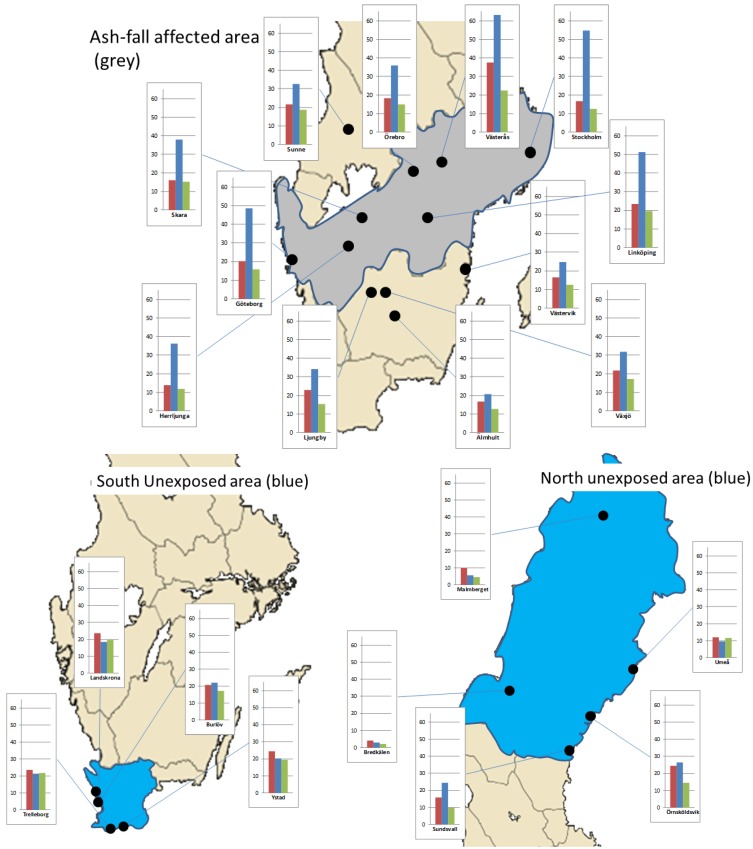
Map of Sweden, shown are the monitoring stations and the Ash area (grey) and the No ash area (blue). The bar diagrams indicate the mean PM_10_ concentrations (µg·m^−3^) four days before (left-red), during the ash fall (middle-blue) and four days after the ash fall (right-green).

A description of the methodologies and sites for ground level hourly mean particle concentrations in Swedish, Norwegian and Finnish cities has been presented elsewhere [1]. Additional data on daily PM ≤ 10 µm in diameter (PM_10_) concentrations were obtained from the Swedish urban air quality network operated by the Swedish Environmental Research Institute (IVL). PM_10_ concentrations were measured gravimetrically by filter sampling as described by Ferm and colleagues [15]. The measurements were made at urban background sites in 14 cities (Burlöv, Herrljunga, Landskrona, Ljungby, Skara, Sunne, Trelleborg, Västervik, Västerås, Växjö, Ystad, Älmhult, Örebro and Örnsköldsvik) and at two rural locations (Bredkälen and Råö; Figure 1). Statistics on mortality and other health indicators are recorded in 21 different administrative regions. Based on the PM_10_ measurements the 21 regions were classified into an Ash area (Stockholm, Uppland, Södermanland, Västra Götaland, Östergötland, Örebro and Västmanland) and a No ash area, (Skåne in the south and Västernorrland, Jämtland, Norrbotten and Västerbotten in the north; Figure 1). The remaining regions (Jönköping, Värmland, Dalarna, Gävleborg, Halland, Blekinge, Kronoberg, Gotland and Kalmar) were excluded from the analysis due to insufficient or contradicting data on particle concentrations.

### 2.2. Exposure Assessment

**Figure 2:**
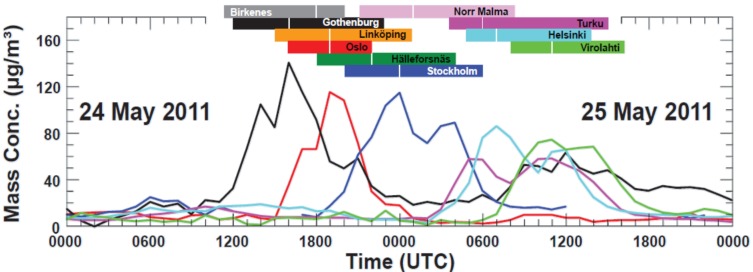
Evolution of coarse particle concentrations (PM_2.5_–PM_10_) in Gothenburg (black), Oslo (red), Stockholm (blue), Turku (magenta), Helsinki (cyan) and Virolahti (green). Colored horizontal bars (include also Linköping (orange), Hälleforsnäs (dark green) and the rural sites Birkenes (gray) and Norr Malma (light magenta)) indicate when concentrations exceed 40 µg·m^−3^. White vertical lines inside the bars shows the time of peak concentrations at each site.

Figure 2 shows the temporal variation of hourly mean PM2.5–10 concentrations in eight cities and at two rural locations in Sweden, Norway and Finland during 24–25 May 2011. Maximum hourly concentrations were in the range 60 to 140 µg·m^−3^. Hourly mean concentrations exceeded 40 µg·m^−3^ during several hours (mainly due to volcanic ash particles) at all measuring sites. In Norway and Sweden, the ash particle event lasted for about 10 h and in Finland for about 7 h. The temporal variations at the different sites reflect the movement of the ash cloud across Norway, Sweden and Finland. The time difference between the peak concentrations in Gothenburg (Sweden) and Helsinki (Finland) was slightly under 24 h. 

The particle fractions of volcanic ash were estimated for different diameters of PM (PM_10_, PM_2.5_ and PM_2.5–10_) from measurements at two rural sites, one on the east- and one on the west coast of Sweden. The distribution of PM fractions was very similar for the two sites. On the west coast the ash particle contributions were 9.8 and 21.0 µg·m^−3^ for PM_2.5_ and PM_2.5_–PM_10_, respectively. On the east coast the corresponding values were 7.4 and 23.1. For simplicity, we estimated the average daily increase of PM_2.5_–PM_10_, to be 20 µg·m^−3^ and the average increase in PM_2.5_ to be 10 µg·m^−3^ in the Ash area on 24–25 May 2011 when calculating the expected number of increased deaths given known dose-response curves.

### 2.3. Mortality Data and Assessment of Impact on Mortality

Data on daily all-cause mortality for residents in each administrative region were obtained from Statistics Sweden for the time period 1 April through 31 July 2011. The exposure period was defined as 24 May through 31 May; alas the week following the two days of elevated particle concentrations, as effects of air pollution on mortality can be delayed several days. A control period was defined as the remaining number of days with available data (1 April through 23 May and 1 June through 31 July). The short exposure period makes conventional time series methods such as case-crossover analysis or Generalized Additive Models non-applicable to analyze data and control for time trends. In order to somewhat control for seasonal trends, that were assumed to be similar across Sweden, we calculated the daily ratios between the number of deaths in the Ash area and the No ash area, using the No ash area as denominator. The sizes of the mortality ratios were then compared between the exposure period and the control period, a higher ratio during the exposure period than during the control period would imply an effect of the ash on mortality.

In a sensitivity analyses the control period was defined in an alternative way, by excluding 21–23 May, when the eruption had begun but before the ash reached Sweden. In another analysis, the exposure period was extended to one month after the ash reached Sweden, from 23 May to 22 June, since effects with up to one month’s delay have been suggested [16].

The estimated extra number of deaths per day during the exposure period was calculated by multiplying the mean number of daily deaths during the entire study period (1 April through 31 July 2011) in the unexposed areas with the change in mortality ratio each day between 24 May and 31 May according to the following Formula (1):

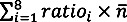
(1)
where *i =* 1 denotes the start of the exposure period (24 May) and *i =* 8, denotes 31 May, *ratio_i_*, denotes the mortality ratio day *i* during the exposure period, and *n* denotes the average number of daily deaths in the No ash area during the whole time period (1 April through 31 July 2011).

## 3. Results

**Figure 3:**
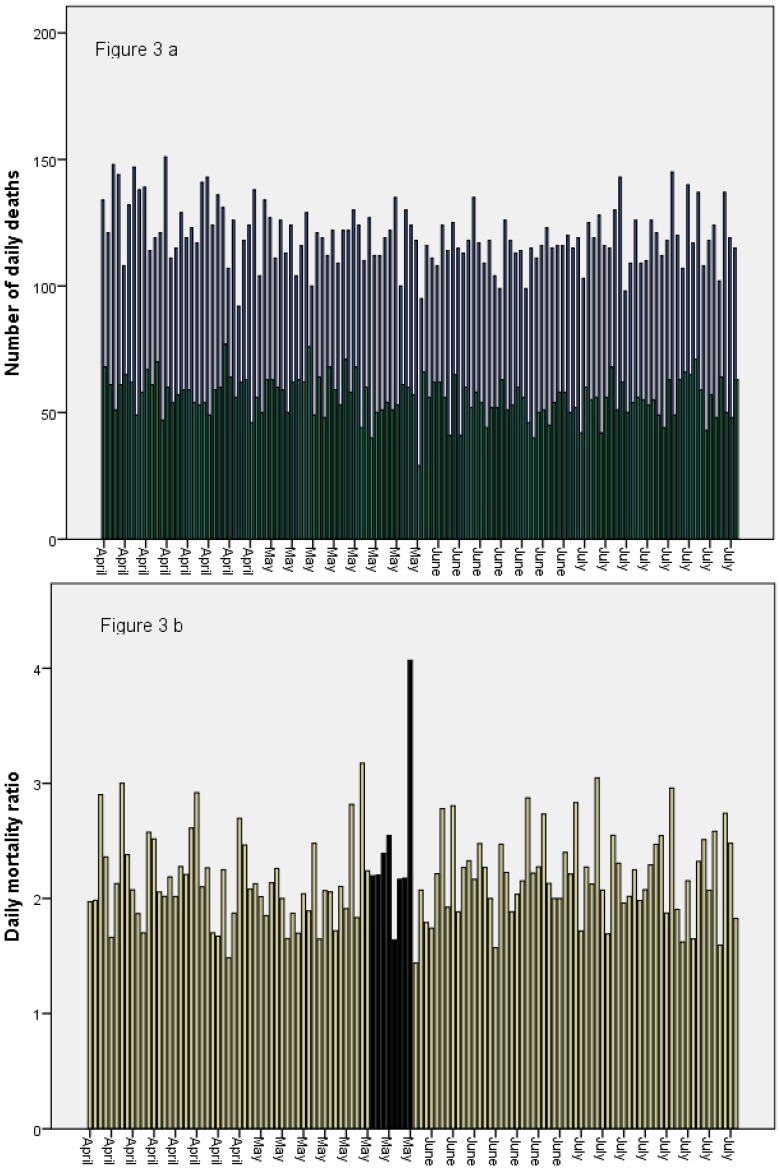
(**a**) Number of daily deaths per day in Ash area (black bars) and No ash area (dark green bars) 1 April–31 July 2011. (**b**) Mortality ratios (The number of daily deaths in the Ash Area divided by the Number of daily deaths in the No ash area) 1 April–31 July 2011. Bars during the exposure period are marked with black.

The mean number of deaths per day during the control period was 120 in the Ash area and 56 in the No ash area. During the exposure period (the week after the ash reached Sweden), the mean number of daily deaths was still 120 in the Ash area, and 52 in the No ash area, a slight decrease (Table 1). In absolute numbers, there was thus no increase in mortality during the exposure period. However, the mortality ratios were higher during the exposure period than during the control period, with a mean ratio of 2.42 during the exposure period compared to a mean ratio of 2.17 during the control period (Table 1, Figure 3a,b). A closer look at the mortality ratios during the exposure period reveals that the increase in mean ratio is caused by an unusually high ratio (4.1) of 31 May, which is the highest ratio during the time period with available data (Figure 3b).

Although the statistical power was low with only 8 days in the exposure period (24 May through 31 May), the differences in mortality ratios were tested with a non-parametric test (Mann-Whitney), and were not statistically significant (*p* > 0.3). However, assuming that the observed differences were not due to chance, it would, according to Formula (1), imply an increase of 128 extra deaths during the exposure period (24 May to 31 May), of which the majority (*n* = 108) occurred on the very last day, 31 May. The additional analysis revealed no indications of any remaining effects in June. The exclusion of 21–23 May from the control period only influenced the results marginally.

**Table 1:** Number of daily deaths and mortality ratios during the exposure period and control period, in the Ash area and in the No ash area.

Outcome	Area	Mean/Median	Exposure Period (24–31 May)	Control Period (1 April–23 May, 1–31 June)
Number of daily deaths	Ash area	Mean	120	120
		Median	121	119
	No ash area	Mean	52	56
		Median	54	56
Mortality ratios ^1^		Mean	2.42	2.17
		Median	2.20	2.13

Note: ^1^ Number of daily deaths in the Ash area divided with the Number of daily deaths in the No ash area.

## 4. Discussion

The results are inconclusive, possibly due to the limited amount of data. There was no increase observed in the absolute number of deaths in the Ash area during the exposure period. However, the relative differences in mortality between the Ash area and the No ash area suggest the additional number of deaths in the Ash area to be 128 during the exposure period, which is surprisingly high compared to known dose-response from urban particulate air pollution. Results from a previous Swedish study suggest that an increase in daily numbers of deaths associated with a 10 µg·m^−3^ the same and previous day (lag01) increase in PM_2.5–10_ was 1.33% in a two-pollutant model [4]. The corresponding increase associated with PM_2.5_ was 0.90% [4]. The expected number of extra deaths given the dose-response observed in Stockholm associated with an average increase in PM_2.5–10_ of 20 µg·m^−3^ and an average increase in PM_2.5_ of 10 µg·m^−3^ (the estimated increase due to volcanic ash 24–25 May) would suggest 1.1 extra deaths due to increased concentrations of PM_2.5_ and 3.2 extra deaths due to PM_2.5–10_ particles on 26 May in the Ash area. Small remaining effects could be expected the following days, but nevertheless, that estimate is substantially lower than suggested by the results of the present study, with an estimated number of extra deaths of 128 during 24–31 May.

The present study has several weaknesses, most notably; the short exposure period yielding a very low inherent statistical power which would only increase marginally by expanding data to cover a longer time period. Moreover, the outcome of the present study is all-cause mortality, which is a rather crude measure since for example accidental mortality surely cannot be attributed to air pollution exposure. Other more specific outcomes such as respiratory symptoms, cardio-respiratory admissions, emergency room visits or age-specific mortality would be more appropriate to study. Unfortunately, data on such outcomes were not available as the present study was undertaken. 

Most of the observed effect seems to be due to an unusually low number of daily deaths in the No ash area on May 31 (Figure 3a,b). If May 31 would be excluded, the number of additional deaths would be 20, which is substantially lower than the estimated 128 for the whole exposure period (24–31 May). By comparing ratios between the exposed and unexposed area, we aimed to control for seasonality, assuming that seasonal trends would not differ between the exposed and unexposed areas. It is important to stress that the absolute number of deaths in the exposed area did not increase during the exposure period, but decreased in the unexposed area, which could be due to seasonal effects. If seasonal trends differed between the exposed and unexposed areas, the method applied in the present study would not be valid. Sweden is a quite large country, and contrasts in for example temperature can be quite large. However, the No ash area consists of both the northernmost and southernmost regions in Sweden, whereas the Ash area is in between, meaning that any differences in seasonal trends between the Ash area and the No ash area might be leveled out. 

As in all air pollution studies on population level, exposure misclassification is certain to occur, which typically leads to an underestimation of the effects [17]. The exposure of the population will differ on time spent outdoors. Also, as exposure is based on residence, part of the population might have been misclassified if they were travelling between the exposed and unexposed areas. Another potential source of error would be if the increased PM concentrations were caused by another potential source than volcanic ash particles, but that seems very unlikely. 

The highest concentrations of PM_10_ recorded at the monitoring sites were caused by PM_2.5–10_ similar to volcanic particles, which consisted of supermicron particles (>1 µm) with peak diameters between 3 and 4 µm [1,2]. PM_1_ mass concentrations or sub-micron particle number size distributions were hardly affected neither in Stockholm [1] nor Helsinki [2]. Individual particle analysis of particles collected in Helsinki revealed the presence of typical volcanic ash particles consisting of basalt rock elements such as Si, Fe, Al, Ca, Mg, Na and Ti, but most supermicron particles were also mixed with sea salt [2]. The plumes of particle-ash and sulphur dioxide were separated shortly after the eruption, explaining why the sulphate concentrations were not particularly elevated. Both in terms of particle size and chemistry, the particles resemble particles typically observed due to traffic-induced suspension of road dust during spring in Scandinavia [18,19,20,21].

The ash cloud passed at night, which minimized the exposure to the population. The maximum ash mass concentrations were estimated to be within the range 150–340 µg·m^−3^ at 2.8 km above ground, based on vertical aerosol LIDAR measurements over Stockholm [1]. Ground level concentrations could have been even higher if the ash cloud had passed during daytime where there is more intense vertical mixing of the atmospheric boundary layer. A study from the United Kingdom indicates that ash concentration and composition can differ quite substantially at adjacent locations [22]. We had no access to exposure data in such detail, but we defined the Ash area and the No ash area as conservatively as possible given available data, and excluded areas where measuring data were insufficient or contradicting. However, some exposure misclassification due to heterogeneity with respect to ash concentration and composition within the exposed and unexposed areas is difficult to avoid in population level studies such as the present study. Such misclassification would likely cause bias towards the null.

The quantity of ash that reached Sweden from the Grimsvötn eruption was relatively small. Future eruptions from Icelandic volcanoes could be of a much larger scale [11]. It is therefore important that even at relatively small ash quantities, there may be substantial effects associated with ash particles compared to the expected effect of particles from more common sources of air pollution, such as traffic. Given the observations of the present study, the potential effects of a larger volcanic eruption could be more severe than previously estimated [11].

The results of the present study warrant further studies in this area. When preparing for a future large-scale eruption in Iceland, it is important to identify sensitive groups in the population. Future studies should focus on groups at particular risk and other outcomes than mortality, but given the short exposure period from the Grimsvötn eruption, most studies will be marred by low statistical power. Meta-analysis or pooled analysis of data from for example all the Scandinavian countries might be necessary to reach sufficient statistical power to study effects of the Grimsvötn ash on morbidity and mortality.

## 5. Conclusions

In this study with limited data we observed contradicting and inconclusive results. We observed no absolute increase in mortality due to the Grimsvötn ash in Sweden, but relative differences in mortality within Sweden suggest a possible ash effect, which was higher than expected given known dose-response curves from urban air pollution. Pooled analysis or meta-analysis with data from other ash affected countries would increase statistical power and thereby increase the possibility of obtaining conclusive results.

## References

[1] Tesche M., Glantz P., Johansson C., Norman M.G., Hiebsch A., Seifert P., Ansmann A., Engelmann R., Althausen D. (2012). Volcanic ash over Scandinavia originating from the Grímsvötn eruptions in May 2011. J. Geophys. Res..

[2] Kerminen V.-M., Niemi J.V., Timonen H., Aurela M., Frey A., Carbone S., Saarikoski S., Teinilä K., Hakkarainen J., Tamminen J. (2011). Characterization of a volcanic ash episode in southern Finland caused by the Grimsvötn eruption in Iceland in May 2011. Atmos. Chem. Phys..

[3] Carlsen H.K., Zoëga H., Valdimarsdóttir U., Gíslason T., Hrafnkelsson B. (2012). Hydrogen sulfide and particle matter levels associated with increased dispensing of anti-asthma drugs in Iceland’s capital. Environ. Res..

[4] Meister K., Johansson C., Forsberg B. (2011). Estimated short-term effects of coarse particles on daily mortality in Stockholm, Sweden. Environ. Health Perspect..

[5] Oudin A., Strömberg U., Jakobsson K., Stroh E., Björk J. (2009). Estimations of short-term effects of air pollution on stroke hospital admissions in Southern Sweden. Neuroepidemiology.

[6] Pope C.A., Muhlestein J.B., May H.T., Renlund D.G., Anderson J.L., Horne B.D. (2006). Ischemic heart disease events triggered by short-term exposure to fine particulate air pollution. Circulation.

[7] Dominici F., Peng R.D., Bell M.L. (2006). Fine particulate air Pollution and hospital admission for cardiovascular and respiratory diseases. JAMA.

[8] Witham C.S., Oppenheimer C. (2004). Mortality in England during the 1783–4 Laki Craters eruption. Bull. Volcanol..

[9] (2012). Smithsonian, Global Volcanism Program: Worldwide Holocene Volcano and Eruption Information. http://www.volcano.si.edu/volcano.cfm?vn=372030.

[10] Thordarson T., Larsen G. (2007). Volcanism in Iceland in historical time: Volcano types, eruption styles and eruptive history. J. Geodynamics.

[11] Schmidt A., Ostro B., Carslaw K.S., Wilson M., Thordarson T., Mann G.W., Simmons A.J. (2011). Excess mortality in Europe following a future Laki-style Icelandic eruption. Proc. Nat. Acad. Sci. USA.

[12] Small C., Naumann T. (2001). The global distribution of human population and recent volcanism. Environ. Hazards.

[13] Hansell A.L., Horwell C.J., Oppenheimer C. (2006). The health hazards of volcanoes and geothermal areas. Occup. Environ. Med..

[14] Horwell C., Baxter P. (2006). The respiratory health hazards of volcanic ash: A review for volcanic risk mitigation. Bull. Volcanol..

[15] Ferm M., Peterson K., Svanberg P.-A., Lövblad G. (2000). Cost-efficient Measurements of PM10 Using Simple Sampling Equipment. EMEP/CCC-Report.

[16] Newnham R.M., Dirks K.N., Samaranayake D. (2010). An investigation into long-distance health impacts of the 1996 eruption of Mt. Ruapehu. Atmos. Environ..

[17] Zeger S.L., Thomas D., Dominici F., Samet J.M., Schwartz J., Dockery D., Cohen A. (2000). Exposure measurement error in time-series studies of air pollution: Concepts and consequences. Environ. Health Perspect..

[18] Norman M., Johansson C. (2006). Studies of some measures to reduce road dust emissions from paved roads in Scandinavia. Atmos. Environ..

[19] Kupiainen K.J., Pirjola L. (2011). Vehicle non-exhaust emissions from the tyre-road interface—Effect of stud properties, traction sanding and resuspension. Atmos. Environ..

[20] Omstedt G., Bringfelt B., Johansson C. (2005). A model for vehicle-induced non-tailpipe emissions of particles along Swedish roads. Atmos. Environ..

[21] Ketzel M., Omstedt G., Johansson C., Düring I., Pohjola M., Oettl D., Gidhagen L., Wåhlin P., Lohmeyer A., Haakana M. (2007). Estimation and validation of PM_2.5_/PM_10_ exhaust and non-exhaust emission factors for practical street pollution modelling. Atmos. Environ..

[22] Stevenson J.A., Loughlin S.C., Font A., Fuller G.W., MacLeod A., Oliver I.W., Jackson B., Horwell C.J., Thordarson T., Dawson I. (2013). UK monitoring and deposition of tephra from the May 2011 eruption of Grimsvotn, Iceland. J. Appl. Volcanol..

